# Plant-Based Diets in Pediatric Subjects: Heart-Healthy Option or Dangerous Choice?

**DOI:** 10.3390/healthcare12222290

**Published:** 2024-11-16

**Authors:** Maria Elena Capra, Delia Monopoli, Brigida Stanyevic, Antonella Giudice, Nicola Mattia Decarolis, Susanna Esposito, Giacomo Biasucci

**Affiliations:** 1 Pediatrics and Neonatology Unit, Guglielmo da Saliceto Hospital, 29121 Piacenza, Italy; m.capra@ausl.pc.it (M.E.C.); g.biasucci@ausl.pc.it (G.B.); 2 Pediatric Clinic, Department of Medicine and Surgery, University of Parma, 43126 Parma, Italy; 3 Department of Medicine and Surgery, University of Parma, 43121 Parma, Italy

**Keywords:** plant-based diet, dietary patterns, children, adolescents, complementary feeding

## Abstract

Background/Objectives: Plant-based diets (PBDs) are dietary patterns characterized by a certain degree of animal-derived food exclusion. PBDs can be divided into different dietary patterns, from vegetarian to vegan, depending on the degree and the extent of animal-derived food avoidance. PBDs are becoming epidemically popular among the general population, including adult subjects as well as children and adolescents, who often follow the dietary pattern chosen by their families. Methods: Our narrative review aims to analyze the most frequently adopted plant-based dietary patterns in children and adolescents and to evaluate their feasibility, advantages, and risks in terms of health promotion and disease prevention in the developmental age. The MEDLINE–PubMed database was searched to collect and select publications from 1980 to 2024. Results: Subjects following these dietary patterns, especially vegan diets, must be under strict nutritional control and receive adequate micronutrients and vitamin supplementation. Conclusions: Nutrition-skilled professionals should be adequately updated and informed about the feasibility and the risks of these different patterns’ adoption at different ages, as they should guide and accompany children and adolescents and their families in their nutritional choices without prejudices, granting adequate macronutrient and micronutrient intake, adequate growth and neurodevelopment.

## 1. Introduction

Dietary patterns characterized by a certain degree of exclusion of animal-derived items have become increasingly popular among adult subjects. In the USA, approximately 6% of adults follow a meat-free diet, and half of them are vegan. Concerning the pediatric population, approximately 3% and 2% of 8- to 17-year-old children follow a non-vegan vegetarian and vegan diet, respectively [1]. These so-called “plant-based diets (PBDs)” comprise a variety of different nutritional habits, which are characterized by various kinds of food avoidance, from the dietary elimination of meat, poultry, and seafood to the intake of exclusively plant-derived foods. Children and adolescents are influenced by the nutritional habits of their families. In most cases, they do not take part in the familial decisional process and they tend to maintain and follow the nutritional habits their parents taught them. While adult subjects have completed their growth and neurodevelopment, before achieving the intended goals, nutritional interventions should always provide sufficient growth and neurodevelopment because children cannot be viewed as little adults. Therefore, dietary patterns that are thought to be heart-healthy for adults may not be appropriate for children.

The purpose of our narrative review is to examine the most commonly used PBDs in children and adolescents and assess their viability, benefits, and drawbacks with regard to illness prevention and health promotion during developmental stages.

We chose to focus only on PBDs considering their increasing diffusion and the impact they may have on food sustainability; however, the novelty of our review is not only this specific focus, but also the analysis of the positive and negative health effects of this dietary pattern throughout the developmental age, starting from toddlers up to adolescents. Indeed, as “children are not little adults”, we believe that an adequate nutritional advice is fundamental to grant them adequate growth and neurodevelopment, while in the meantime respecting their habits and traditions. The MEDLINE–PubMed database was searched to collect and select publications from 1980 to 2024. The search included randomized placebo-controlled trials, controlled clinical trials, double-blind, randomized controlled studies, and systematic reviews. The following combinations of keywords were used: “plant-based diet” OR “plant-based dietary pattern” OR “vegetarian” OR “vegan” AND “children” OR “adolescents” OR “pediatric” AND “growth” OR “advantages” OR “risk” OR “complementary feeding”. We also performed a manual search of the reference lists of the selected studies. The search was limited to English-language journals and full papers only.

## 2. Plant-Based Dietary Patterns

There are several types of PBDs, each one characterized by its level of exclusion of animal products. Among these dietary patterns, the vegan diet, characterized by the exclusion of all animal-derived foods from the diet (meat, fish, and animal derivatives) is one of the most restrictive. Other diets include the lacto-ovo vegetarian, in which eggs and dairy products are allowed, the lacto-vegetarian and ovo-vegetarian diets, allowing dairy products and eggs intake, respectively, with the exclusion of any other animal-based foods, and the pescetarian diet, which permits fish intake. Additionally, there are more stringent variants, such as the raw food diet, characterized by the avoidance of cooked food, dairy products, eggs, and meat, the macrobiotic diet, which emphasizes the consumption of whole grains, algae, vegetables, and legumes while restricting sugar, refined foods and animal products, and the fruitarian diet, which is based on the consumption of fruits, vegetables and nuts [1,2]. The main PBDs are summarized in Table 1.

While plant oils are a great source of polyunsaturated fatty acids (PUFAs), in particular the essential fatty acids α-linolenic acid (ALA) and linoleic acid (LA), which are vital for pediatric subjects’ growth and development, plant foods are high in fiber and vitamins C and E [3]. It has been proposed that dietary patterns that provide an adequate energy requirement and include a variety of nutrient-dense plant foods may meet the dietary requirements for protein and most micronutrients, even though the bioavailability of certain nutrients may be limited predominantly in PBDs, primarily in vegan diets [1]. The number of PBD adherents is steadily rising, particularly in wealthy nations. PBDs can be regarded as more sustainable because of the growing evidence that environmental degradation caused by greenhouse gas emissions and other pollution agents, as well as the fact that the amount of resources used to produce plant-based foods is substantially lower than that of animal-based foods [1].

In the United States of America, according to the National Health and Nutrition Examination Surveys (2007–2010), 2.1% of adults follow a vegetarian diet [4]. In Europe, the percentage of people following vegetarian diets varies according to geographical areas, with an estimated 1.2% to 1.5% in Portugal and Spain, 7% in the United Kingdom, and 10% in Germany [5]. Based on these data, we might speculate that PBDs are also becoming more widespread among adolescents and children, who are completely dependent on their family’s habits and traditions.

According to the recent KiGGS study conducted in Germany, 3.4% of children and adolescents adhered to a vegetarian (including vegan) diet between 2015 and 2017, with a constant increase since 2006 [6]. Indeed, over the past decade, the number of vegan subjects in Germany has shown a 15-fold increase, rising from 0.1 million in 2012 to 1.58 million in 2022 [7]. Based on the 2010 UK National Diet and Nutrition Survey (NDNS), 2% of adults and children identified themselves as vegetarians, whereas less than 1% adhered to a vegan diet [7]. A recent Italian study reported that nearly 9% of Italian infants are weaned following a vegetarian or vegan schedule. Moreover, nearly half (45.2%) of parents could not obtain suitable dietary guidance from their pediatricians, as family doctors often lacked expertise in this area [8].

People may choose a vegetarian diet for several reasons, including ecological, economic, religious, ethical, and health-related factors. As far as environmental issues are concerned, it should be considered that vegan and vegetarian diets have significantly lower greenhouse gas emissions, and land and water use requirements compared to meat-containing diets. Additionally, PBDs tend to be healthier, with a lower environmental impact compared to animal-based foods diets [9]. This may be due to direct processes related to livestock management [such as methane (CH4) production by ruminants], and indirect processes involving the inefficiency of using crops for animal feeding, instead of direct human consumption [10]. Consequently, proposed diets for global sustainable food production recommend that adult subjects from high-income countries significantly reduce their consumption of animal-based foods, aligning it with the current much lower intake in many low-income countries [11].

Health-related issues can influence people to follow a PBD as well, to lose weight, improve physical fitness, and reduce risk for cardiovascular diseases, diabetes, and cancer [12]. Additionally, in populations like Adventists and Mormons, which follow a PBD for specific religious beliefs, lower blood pressure values have been described [13]. The practice of vegetarianism is strongly linked with several religious traditions worldwide, including Hinduism, Jainism, Buddhism, and Sikhism. In a study conducted in 2021, the authors analyzed the various motivations that led a cohort of 511 adult subjects to adopt a vegetarian diet: 43% chose a vegetarian diet due to animal rights, 31% cited health reasons, and 24% mentioned other factors [14]. However, despite this evidence of PBD-related health benefits in adult subjects, data available on PBDs nutritional safety in children are still limited. Indeed, most available data are derived from studies on adults, and many of the existing studies on children are quite old-fashioned and focus solely on vegetarian subjects [1].

## 3. Plant-Based Dietary Patterns and Complementary Feeding

Nutritional intake during the so-called first thousand days from conception plays a crucial role in long-term adult health, affecting weight growth, neurodevelopment, nutritional preferences, body weight, and the predisposition to the development of non-communicable diseases [15]. Complementary feeding (CF) is defined as the process of introducing solid or liquid foods (complementary foods—CFs), other than human breast milk (HBM) or infant formula, into the human diet when HBM or infant formula is no longer sufficient to meet infants’ nutritional needs. CF is one of the most important and crucial time windows in human development, in which a positive or negative epigenetic intervention may also have relevant effects in later life [15].

Recently, a 6-month longer breastfeeding duration has been reported in infants on vegetarian or vegan diets compared to those following an omnivorous diet (15.8 months vs. 9.7 months) [8]. Prolonged breastfeeding may further enhance the risk of specific micro- and macronutrient deficiencies in infants following PBDs during CF. Potential deficiencies may include protein, calcium, vitamin D, vitamin B12, iodine, zinc, iron, and omega-3 fatty acids [16,17]. Consequently, scientific societies have expressed their opinion worldwide on the feasibility and possible risks of adopting PBDs during CF.

In a recent position paper, the European Society of Pediatric Gastroenterology, Hepatology, and Nutrition (ESPGHAN) examined various facets of CF with an emphasis on healthy term European infants aged 4–12 months. The scientists came to the conclusion that proper growth and development may be supported by vegetarian meals supplemented with the right nutrients. In any case, to guarantee dietary nutritional adequacy, routine medical and dietary supervision is essential. Indeed, self-planned dietary patterns may lead to severe consequences, including irreversible cognitive impairment, and death. The vegan diet is not recommended during the first two years of life, but in case of irreversible parental choice, the position paper stresses the importance of carefully monitoring infants and young children, with the need for regular medical and dietary supervision [18].

Mangels et al. [19] outlined a timeline for introducing solid foods for vegetarian and vegan infants. Between four and six months, iron-fortified infant cereals should be the first solid foods to be introduced, as they offer adequate energy, and iron in an easily digestible form. Once the infants tolerate cereals, vegetables, and fruits can be added in any order. From seven to eight months, protein sources should be incorporated. Suitable protein options for vegetarian infants include soy yogurt, tofu, and legume puree. Tempeh and soy burger introduction is recommended for around 11 to 12 months [19,20]. A well-planned vegan diet can meet current nutritional demands and is suitable for all periods of life, including pregnancy, lactation, infancy, childhood, adolescence, and maturity, according to Canadian dietitians in 2003, who agreed with the American Dietetic Association [21]. The Canadian Pediatric Society did not voice a negative opinion regarding PBDs during CF, but they did emphasize the significance of keeping an eye on children’s protein and calorie intake and supplementing their meals when needed. [22].

The 2020–2025 Dietary Guidelines for Americans provide recommendations for a healthy vegetarian eating pattern for children. For those aged 12–24 months, who do not consume meat, poultry, or seafood, it is essential to regularly include eggs, dairy products, soy-based products, nuts, seeds, fruits, vegetables, and oils in their diets [23].

A well-planned vegetarian diet can be a healthy substitute for all phases of fetal, infant, child, and adolescent development, according to a statement made by the American Academy of Pediatrics fifteen years ago. Further noteworthy is the position taken on vegan formulas by the North American Society for Pediatric Gastroenterology, Hepatology, and Nutrition (NASPGHAN). This is particularly important when a dairy-free diet is followed for the first year of life and breastfeeding is not an option. As appropriate substitutes for formulas made with cow’s milk, NASPGHAN emphasizes the significance of appropriately supplemented vegan formulas, such as those made from soy or rice [24]. Conversely, a study conducted a decade ago on a single family consisting of two parents and their two children (a 12-year-old boy and a 10-month-old girl) who were on a vegan diet revealed that both children experienced anemia and developmental delay as a result of vitamin B12 deficiency because their mother followed a strict vegan diet during her pregnancy and lactation without receiving any special vitamin supplements or nutritional counseling [25]. Pregnant women and children may be at risk from vegan diets, according to a 2019 report by the Royal Academy of Medicine of Belgium, an advisory group for Belgian government institutions with scant citations. The committee concluded that vegan diets were unsuitable and did not advise pregnant and lactating women, children, or teenagers to follow them [26].

A vegan diet is “not recommended for infants, children, and adolescents due to the inevitable risk of multiple nutritional deficiencies without supplementation,” according to the French-speaking Pediatric Hepatology, Gastroenterology, and Nutrition Group [27].

The feasibility of PBDs in childhood has been evaluated and discussed in the joint position paper of the Italian Society of Preventive and Social Pediatrics (SIPPS) with the Italian Federation of Pediatricians (FIMP) and the Italian Society of Perinatal Medicine (SIMP). The authors conclude that vegan dietary patterns should not be recommended for children until two years of age due to the possible macro and micronutrient deficiency linked to these nutritional habits and consequent growth and neurodevelopment delay [28].

Some perspectives aim to find a middle ground, seeking a compromise between ensuring adequate calorie intake during weaning, and parents’ preference for vegetarian or vegan diets. A diversified and well-balanced ovo-lacto-vegetarian diet can satisfy young children’s and adolescents’ energy and nutrient needs, according to the German Nutrition Society (DGE). Adolescents should not completely stop eating animal products as a permanent dietary pattern, according to the DGE [29]. In addition to advocating for required vitamin B12 supplementation and ongoing monitoring of nutrients at risk in vegan children, the Spanish Pediatric Association [30] recommends omnivore and lacto-ovo-vegetarian diets for infants and young children rather than vegan diets.

Kostecka et al. [5] assessed the nutritional knowledge of plant-based or omnivorous diets of parents raising 12- to 36-month-old children. The authors asked 524 women to fill in a questionnaire investigating their nutritional habits: a total of 146 women (44.8%), 48 vegans (14.7%), 64 ovo-vegetarians (19.6%), 68 lacto-vegetarians (20.9%), and 198 omnivores maintained their children on a lacto-ovo-vegetarian diet. With an average score of 15.8 points on the assessed scoring system, the examination of these questionnaires revealed that mothers who fed their kids a lacto-ovo-vegetarian diet had a deeper understanding of nutrition. Conversely, mothers in the control group, and those raising children on a vegan diet had the lowest scores, with an average score of 13.8 points. The results of this study highlighted that both types of diet and maternal nutritional knowledge can influence the dietary patterns proposed to children aged 12 to 36 months. Mothers who followed more restrictive vegetarian diets were more aware of the risk of nutritional deficiencies, and more frequently administered dietary supplements. Indeed, children on a vegan diet are particularly vulnerable to nutritional deficiencies, resulting from the inadequate intake of essential nutrients, and/or high consumption of fiber and other plant-based components that lower nutrient bioavailability [31]. Vitamin C contained in vegetables and fruits enhances the absorption of non-heme iron [32], although some plant compounds can inhibit it. Parents should be informed about iron-rich foods, and include them in their children’s diets, especially iron-fortified cereals, dried beans, and peas, as iron deficiency is the most common nutritional disorder in children, both omnivorous and vegetarian [31].

In conclusion, a vegetarian diet may be safe for young children even during CF. The decision to start a vegetarian or vegan weaning should be made under close medical monitoring, to avoid potential and detrimental nutritional deficiencies. Vegetarian CF, if conducted under medical supervision, is feasible and should not be hampered. Vegan CF, on the other hand, should be carefully evaluated, considering that serious health-threatening conditions, such as failure to thrive, rickets, and neurodevelopment delay, have been related to vegan CF. Currently, based on the available evidence, vegan CF is not recommended by the main international scientific institutions. In any case, when choosing a non-omnivorous CF, parents, and caregivers have to be informed about the risks of nutritional deficiencies and the principles of healthy nutritional habits, regardless of the dietary pattern followed. Clear communication between parents, pediatricians, and dietitians is essential, and should lay the foundation of any nutritional strategy for managing vegetarian children.

## 4. Plant-Based Dietary Patterns for Children and Adolescents

The health benefits related to vegan dietary patterns in adults are not confirmed in children and adolescents. Available evidence often relies on outdated, unrepresentative, or information-limited studies on vegan diets, support systems, and specific dietary choices. Additionally, vegan diets are often poorly structured and lack supplementation of vitamins, such as vitamin B12. Similarly, there is no basis for claiming that an omnivorous diet is inherently superior, optimal, or a gold standard for pediatric nutrition. The adoption of a rigorous and unbiased approach when evaluating both vegan and omnivorous diets can help parents and healthcare professionals achieve the information and guidance, they need to make informed and conscious decisions about infant feeding [33].

In a systematic review, Schürmann et al. [34] analyzed PBDs in children, highlighting the lack of adequate studies for this age category. Evaluating relevant studies published in Germany and England up to November 2014, the authors found that it is not possible to reach definitive conclusions about the effects of PBDs on children. While no harmful effects were found, some risks were identified concerning nutrient deficiencies, particularly iron and vitamin B12. Furthermore, more in-depth studies should evaluate less-considered nutrients, such as omega-3 fatty acids, and iodine. On the other hand, PBDs appear to have a positive effect on improving lipid profiles, reducing the risk of overweight, and increasing fiber intake [15]. In 2022, Elliot et al. [35] conducted a longitudinal cohort study aimed at assessing the effects of a vegetarian diet on growth, lipid profile, and micronutrient intake of 8907 children (248 vegetarians), aged 6–8 years. The authors found no significant differences in growth or nutritional status between children on vegetarian diets and those on non-vegetarian diets. However, vegetarian children were more likely to be underweight. This finding highlights the importance of careful and personalized dietary planning for vegetarian children, especially those at risk of underweight [35]. A Finnish cross-sectional study further supports the similar growth and weight outcomes between vegetarian and omnivorous children. This study, involving 40 children (10 vegetarians, 6 vegans, and 24 omnivores) with an average age of 3.5 years, highlighted differences in micronutrient intake according to the dietary patterns followed. Vegan children had a higher intake of fiber, folate, iron, and zinc without supplementation, but a lower intake of protein and saturated fatty acids. Their lipid profile was also more heart-healthy if compared to that of omnivorous children [36]. Improving lipid profile is a crucial factor in cardiovascular risk prevention. Foods like fruits, vegetables, pulses, whole grains, nuts, and oilseeds are rich in unsaturated fatty acids and micronutrients such as phytonutrients and vitamins. These foods have low energy density and are low in saturated fats. Replacing saturated fats with unsaturated fats can favor lower Low-Density Lipoprotein (LDL) cholesterol levels. In addition, adequate dietary fiber intake reduces intestinal absorption of cholesterol and reabsorption of bile acids, with an overall lowering effect on cholesterol synthesis and a further decrease in LDL cholesterol plasma levels. However, most studies have been conducted on the adult population, and only a few studies have been conducted on a pediatric sample [37].

A pilot randomized controlled trial conducted in Cleveland, Ohio, compared the effects of a whole-foods, plant-based diet with an American Heart Association (AHA) guideline-based diet in 30 obese children with hypercholesterolemia and their parents. Both groups showed improvements in the tested items, but those belonging to the vegetarian group showed significantly greater BMI reduction, body fat reduction, better systolic blood pressure, lower total cholesterol, LDL cholesterol, insulin, and waist circumference, and increased fiber intake. The intake of vitamin D, omega-3 fatty acids, calcium, and vitamin B12 has been strictly monitored in both groups, and they both experienced a decrease in these nutrient intakes [38]. In 2021, Macknin et al. [39] conducted a 52-week prospective, randomized trial to evaluate changes in cardiovascular disease risk markers associated with PBDs, the Mediterranean Diet, and the AHA diet, in 96 patients with obesity and hypercholesterolemia, aged 9–18 years. The authors concluded that LDL-C and total cholesterol plasma levels, weight, and systolic and diastolic blood pressure were reduced in all groups, with no statistically significant differences [39].

One of the most common vitamin deficiencies among subjects following a PBD is vitamin B12 deficiency. Vitamin B12 is an essential nutrient for the functioning of the nervous system, the production of red blood cells, and DNA synthesis. While vitamin B12 is naturally present in some animal-based foods, such as meat, fish, eggs, and dairy, completely plant-based or vegan diets are naturally devoid of it. A cross-sectional study involving 187 Polish children aged 5–10 years revealed that the risk of vitamin B12 deficiency was higher in vegetarian children but was reduced in the group supplemented with vitamin B12. This highlights the importance of proper planning and supplementations in PBDs [40,41]. Vegan mothers who do not take vitamin B12 supplementation and breastfeed their infants can provoke a vitamin B12 deficiency in their infants as well, leading to long-lasting neuro-disability and neurological impairment even if vitamin B12 deficit is restored [42,43]. Vitamin B-12 deficiency is a real hazard in unsupplemented or unfortified vegan diets for all children and adolescents [44].

Calcium can be deficient in plant-based dietary patterns, especially in vegan diets. Some vegetable sources provide absorbable calcium, but the quantity of vegetables required to reach adequate calcium intake makes an exclusively plant-based diet impractical for most individuals unless fortified foods or supplements are included [45].

Zinc is another mineral that can be deficient in vegan diets, as its bioavailability may be reduced by the high contents of phytic acid and dietary fiber, and the low content of flesh foods in the diet. Indeed, Gibson analyzed zinc bioavailability in subjects following the vegan diet, showing adequate serum concentration of this trace element in most adult subjects following this dietary pattern. Children, however, are more vulnerable to suboptimal zinc status, presumably because of their high zinc requirements for growth and their bodies’ failure to adapt to a vegan diet by increased absorption of dietary zinc [46].

Possible micronutrients and vitamin deficiencies and proposed supplementations in infancy, childhood, and adolescence are summarized in Table 2.

## 5. Conclusions

Plant-based dietary patterns are very popular worldwide and include a variety of very different nutritional patterns. Subjects following these dietary patterns, especially the vegan diet, must be under strict nutritional control, and receive adequate micronutrient and vitamin supplementation. PBDs are often started without precise nutritional planning. This is a health risk, as micronutrients and macronutrients excess and/or deficiency, as well as their effects on health, should be foreseen and avoided while also using adequate supplementation, or fortified foods. In this context, further and more consistent data from longitudinal studies are needed to better assess the PBD effect on health in the developmental age, considering the increasing number of families which adhere to these diets. Children and adolescents who follow plant-based dietary patterns, either induced by their families or following personal decisions, should receive close growth and neurodevelopment monitoring. Nutrition-skilled professionals should be adequately updated and informed about the feasibility and the risk of these different dietary patterns’ adoption at different ages, and they should guide and accompany children and adolescents and their families in their nutritional choices without prejudices, granting adequate macro- and micronutrient intake, as well as adequate growth and neurodevelopment.

## Figures and Tables

**Table 1:** Different types of PBD patterns.

Vegan diet	The most restrictive diet; all animal products, including meat, fish, dairy, eggs, and honey are excluded. Vegans base their diet on a wide variety of plant foods, including fruits, vegetables, legumes, whole grains, nuts and seeds
Lacto-ovo-vegetarian diet	Meat, fish, and seafood are excluded, but dairy products and eggs are permitted. Lacto-ovo vegetarians have a generally more varied diet than vegans and can include foods such as yogurt, cheese, milk, butter, and eggs.
Ovo-vegetarian diet	Meat, fish, seafood, and dairy products are excluded, but egg intake is permitted in any way: boiled, scrambled, fried, or used to make cakes and other dishes.
Lacto-vegetarian diet	Meat, fish, seafood, and eggs are excluded, but dairy products are permitted. Lacto vegetarians can include foods such as yogurt, cheese, milk, and butter in their diet.
Pescetarian diet	Meat, poultry, and seafood are excluded, but fish intake is permitted. Pescetarians choose not to eat meat for various reasons, including health concerns, the ethics of animal treatment, or the environmental impact of animal agriculture.
Raw vegan diet	Only raw or partially cooked food is permitted, such as fruits, vegetables, nuts, seeds, and some sprouts. Raw foodists avoid cooked foods, dairy products, eggs, and meat.
Macrobiotic diet	This diet is based on the philosophical principles of Yin and Yang and aims to create balance in the body. Macrobiotics consume mainly whole grains, legumes, vegetables, and algae while limiting the consumption of meat, dairy products, eggs, sugar, and refined foods.
Fruitarian diet	Mostly based on fruits, vegetables, and nuts intake. Fruitarians may also include seeds and sprouts, but they avoid grains, legumes, dairy products, eggs, and meat.

**Table 2:** Micronutrients and vitamin deficit in plant-based diets and suggested supplementation according to LARN [47].

Type of PBD	Nutritional Risks and Clinical Complications in Infants	Nutritional Risks and Clinical Complications in Children and Adolescents	Recommendations
Vegan diet	Vitamin B12 deficit → neurodevelopmental deficit, anemia Vitamin D deficit → bone development impairment Iron deficit → Anemia, higher susceptibility to infections Zinc deficit → acrodermatitis DHA deficit → visual and cerebral complications	Vitamin B12 deficit → neurodevelopmental deficit, anemia Vitamin D deficit → bone development impairment Iron deficit → Anemia, higher susceptibility to infections Zinc deficit → acrodermatitis DHA deficit → visual and cerebral complications	Vitamin B12 supplementation Age 0–12 months 0.7 µg/day Age 1–3 years 0.9 µg/day Age 4–6 years 1.1 µg/day Age 7–10 years 1.6 µg/day Age 11–14 years 2.2 µg/day Age 15–17 years 2.4 µg/day Folic acid supplementation Vitamin D supplementation Age 0–17 years 15 µg/day Iron supplementation according to Hb and ferritin plasma values DHA 100 mg/day
Vegetarian Diet	No risks if adequately planned and if all food items are consumed in a varied diet	No risks if adequately planned and if all food items are consumed in a varied diet	
Pescetarian	Risk of calcium deficit if no consume of milk and dairy products	Possible calcium supplementation	Possible calcium supplementation
Raw vegan diet	All the risks listed for a vegan diet Risk of intestinal infections due to uncooked food consumption	All the supplementations recommended for a vegan diet	All the supplementations recommended for a vegan diet
Macrobiotic Diet	All the risks listed for a vegan diet	All the supplementations recommended for a vegan diet	All the supplementations recommended for a vegan diet
Fruitarian Diet	All the risks listed for a vegan diet	All the supplementations recommended for a vegan diet	All the supplementations recommended for a vegan diet

## Data Availability

Not applicable.

## Abbreviations

AHA	American Heart Association
CF	Complementary Feeding
ESPGHAN	European Society of Pediatric Gastroenterology Hepatology and Nutrition
LDL-C	Low-Density Lipoprotein Cholesterol
NASPGHAN	North American Society for Pediatric Gastroenterology, Hepatology, and Nutrition
NDNS	National Diet and Nutrition Survey
PBD	Plant-Based Diets
PUFAs	Polyunsaturated Fatty Acids
